# A case report of neuronal intranuclear inclusion disease with paroxysmal peripheral neuropathy-like onset lacking typical signs on diffusion-weighted imaging

**DOI:** 10.3389/fneur.2023.1117243

**Published:** 2023-02-10

**Authors:** Jiayu Fu, Chen Zhao, Guanghao Hou, Xiaoxuan Liu, Mei Zheng, Yingshuang Zhang, Shuo Zhang, Danfeng Zheng, Yixuan Zhang, Xiao Huang, Shan Ye, Dongsheng Fan

**Affiliations:** ^1^Department of Neurology, Peking University Third Hospital, Beijing, China; ^2^Beijing Municipal Key Laboratory of Biomarker and Translational Research in Neurodegenerative Diseases, Beijing, China; ^3^Department of Pathology, Peking University Third Hospital, Beijing, China

**Keywords:** neuronal intranuclear inclusion disease, peripheral neuropathy, NOTCH2NLC gene, skin biopsy, neurodegenerative disease

## Abstract

**Background:**

Neuronal intranuclear inclusion disease (NIID) is a slowly progressive neurodegenerative disease characterized by eosinophilic hyaline intranuclear inclusions and the GGC repeats in the 5'-untranslated region of *NOTCH2NLC*. The prevalent presence of high-intensity signal along the corticomedullary junction on diffusion-weighted imaging (DWI) helps to recognize this heterogeneous disease despite of highly variable clinical manifestations. However, patients without the typical sign on DWI are often misdiagnosed. Besides, there are no reports of NIID patients presenting with paroxysmal peripheral neuropathy-like onset to date.

**Case presentation:**

We present a patient with NIID who suffered recurrent transient numbness in arms for 17 months. Magnetic resonance imaging (MRI) showed diffuse, bilateral white matter lesions without typical subcortical DWI signals. Electrophysiological studies revealed mixed demyelinating and axonal sensorimotor polyneuropathies involving four extremities. After excluding differential diagnosis of peripheral neuropathy through body fluid tests and a sural nerve biopsy, NIID was confirmed by a skin biopsy and the genetic analysis of *NOTCH2NLC*.

**Conclusion:**

This case innovatively demonstrates that NIID could manifest as paroxysmal peripheral neuropathy-like onset, and addresses the electrophysiological characteristics of NIID in depth. We broaden the clinical spectrum of NIID and provide new insights into its differential diagnosis from the perspective of peripheral neuropathy.

## Introduction

Neuronal intranuclear inclusion disease (NIID) is a rare neurodegenerative disease with a wide range of clinical manifestations, including dementia, movement disorder, muscle weakness, and autonomic dysfunction (1). Multiple organs are injured in NIID with the pathological presence of eosinophilic hyaline intranuclear inclusion bodies in cells of nervous system, visceral organs, and skin (2). Characteristic hyperintense areas in the corticomedullary junction on diffusion-weighted imaging (DWI) in brain magnetic resonance imaging (MRI) facilitates its recognition (3). In recent years, diagnosis of NIID is confirmed by an abnormal GGC repeat expansion of the *NOTCH2NCL* gene or a positive skin biopsy with characteristic pathology (4). Whereas paroxysmal symptoms were reported in NIID, the episodes were restricted to encephalopathy with characteristic brain imaging findings (5). In this report, we present a case of NIID exhibiting paroxysmal peripheral neuropathy-like onset before the emergence of typical subcortical DWI signals. The patient's clinical manifestations are unique for NIID and the subclinical electrophysiological changes could be prevalent in the disease. His diagnosis was confirmed by a skin biopsy and genomic analysis.

## Case presentation

**Figure 1:**
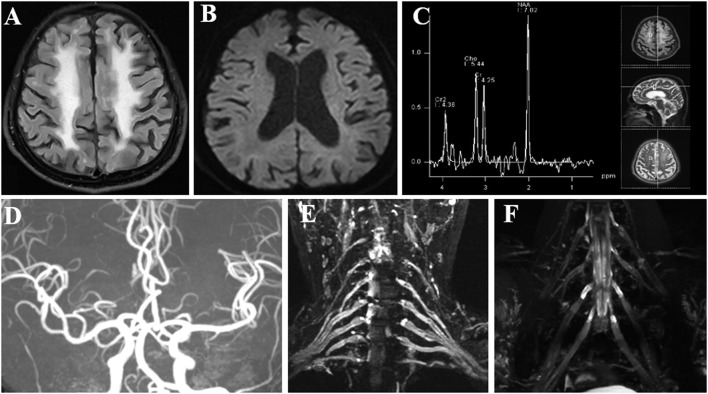
Imaging examinations of the patient. **(A)** FLAIR sequence shows diffuse, bilateral white matter lesions with hyperintensity. **(B)** DWI sequence shows no characteristic hyperintense areas in the corticomedullary junction. **(C)** MRS focused on the frontal lesion shows no lactate peak appearance. **(D)** MRA shows no significant intracranial aortic stenosis or occlusion changes. **(E, F)** Brachial and lumbosacral plexus MRI showed extensive thickening in the nerve roots. FLAIR, fluid-attenuated inversion recovery; DWI, diffusion-weighted image; MRS, magnetic resonance spectroscopy; MRA, magnetic resonance angiography; MRI, magnetic resonance imaging.

**Figure 2:**
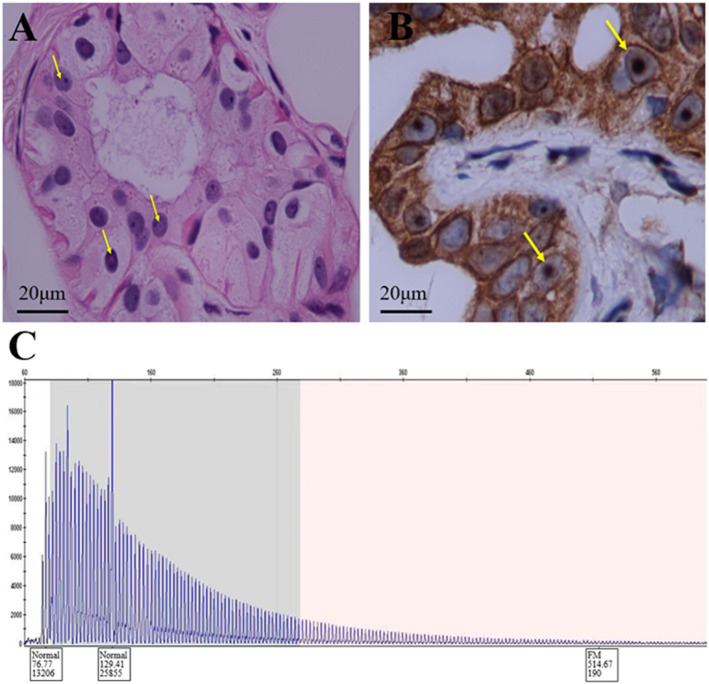
Histopathologic and genetic analyses of the patient. **(A)** Hematoxylin-eosin staining shows eosinophilic inclusion bodies in some sweat gland cells. Scale bars 20 μm. **(B)** Anti-P62 immunohistochemical staining. Visible inclusion bodies in the nucleus of some skin sweat gland cells. Scale bars 20 μm. **(C)** RP-PCR of the patient with a characteristic saw-tooth pattern for an expansion of 151 GGC repeated in the 5′-untranslated region of the NOTCH2NLC gene. RP-PCR, repeat-primed PCR.

A 60-year-old retired driver was admitted to our hospital because of recurrent transient numbness in his arms for 17 months in July 2022. The attacks, which lasted 10–30 min, initially occurred once or twice a month and involved the distal upper extremities from fingers to elbows. A year later the involved site spread to his face with similar numbness around the lips. However, the frequency and duration of the disturbance remained. During these attacks, the patient denied limb weakness, awareness impairment, epilepsy, headache, or other neurological defects. His daughter noticed a progressive cognitive decline manifested as recent amnesia and apathy which mildly limited activities of daily living. The insidious onset of his cognitive dysfunction was around a year ago when he began to forget the issues mentioned through phone calls. However, the patient could complete housework independently and had never lost his way so far. The patient had a history of macular degeneration for 30 years and was diagnosed with ischemic colitis when he was 59. His family had a hereditary history of essential tremor including the patient, his sister, father, and grandmother. His father was diagnosed with dementia of unknown etiology at the age of 60 and died of lung cancer in his eighties. No family history displayed neurodegenerative diseases or peripheral neuropathies. Neurologic examination demonstrated comprehensive cognitive decline including memory impairment, disorientation and attention disturbances, visuospatial dysfunction, language problem, and execution dysfunction. Miosis, static and postural tremors of hands, ataxia, reduced tendon reflexes, and bilateral hypoesthesia were noted without obvious muscle weakness or autonomic dysfunction. Mini-Mental State Examination and Frontal Assessment Battery were 19 and 8, respectively. The results of routine laboratory tests involving autoantibodies, biochemistry, and metabolism were within normal limits. The serum IgM level was elevated to 325 mg/dl with IgM-kappa–type M-protein identified by serum immune electrophoresis. However, no hematological cancer was identified through the biopsy of bone marrow. An increase of CYFRA21-1 (8.67 ng/ml) was noted and the patient was subsequently diagnosed with early-stage lung cancer through the related biopsy in the operation. Analysis of cerebrospinal fluid revealed increases in protein (126.5 mg/dl) and glucose (4 mmol/l) levels with a normal cell count. No abnormalities were found in either blood or cerebrospinal fluid autoantibody tests of peripheral neuropathy, including anti-ganglioside antibodies (GM1, GD1b, GQ1b), para-nodal antibodies (CNTN1, NF155, NF186, CASPR1), and paraneoplastic antibodies (Hu, Yo, Ri, CV2, Ma2, amphiphysin, GAD65). Brain MRI showed diffuse, bilateral white matter lesions without hyperintensity in the white matter adjacent to the cortex on DWI (Figures 1A, B). However, strokes or mitochondrial diseases were unsupported by negative findings in the magnetic resonance angiography and the magnetic resonance spectroscopy (Figures 1C, D). Brachial and lumbosacral plexus MRI showed extensive thickening in the nerve roots (Figures 1E, F). Electrophysiological studies revealed mixed demyelinating and axonal sensorimotor polyneuropathies, and the severity of those objective impairments in nerves were far beyond the clinical symptoms (Table 1). Motor and sensory nerve conduction velocity extensively decreased in the four extremities of the patient with relatively normal compound muscle action potentials (CMAP), while sensory nerve action potentials (SNAP) were absent in bilateral sural nerves. Prolonged latency of ulnar F-waves and tibial H-reflexes were noted. Neither pathological findings of a right sural nerve nor whole-exome sequencing suggested any hereditary peripheral neuropathies or inherited leukoencephalopathies. Therefore, we performed a skin biopsy and found eosinophilic inclusions positive for anti-p62 in the fibroblasts and sweat gland cells of the skin tissue (Figures 2A, B). Additionally, an expansion of 151 GGC repeated in the 5′-untranslated region of the *NOTCH2NLC* gene was detected by repeat-primed polymerase chain reaction (Figure 2C), further confirming the diagnosis of NIID.

**Table 1:** Summary of electrophysiological data.

	**Left**			**Right**		
**Motor**	DML, ms	CMAP, mv	CV, m/s	DML, ms	CMAP, mv	CV, m/s
Median nerve	4.71 (<4.1)	6.5 (>4.0)	29.9 (>40)	5.02 (<4.1)	7.5 (>4.0)	35.4 (>40)
Ulnar nerve	8.54 (<3.2)	5.8 (>4.0)	35.9 (>40)	9.14 (<3.2)	7.2 (>4.0)	35.1 (>40)
Peroneal nerve	13.8 (<5.3)	3.7 (>2.0)	41.1 (>40)	14.7 (<5.3)	4.2 (>2.0)	3.67 (>40)
Tibial nerve	15.8 (<5.0)	7.1 (>4.0)	35.7 (>40)	17.9 (<5.0)	7.5 (>4.0)	31.2 (>40)
**Sensory**	DML, ms	SNAP, μv	CV, m/s	DML, ms	SNAP, μv	CV, m/s
Median nerve	2.71	9.8 (>5.0)	33.2 (>40)	2.95	10.3 (>5.0)	30.5 (>40)
Ulnar nerve	3.06	6.1 (>5.0)	32.7 (>40)	2.69	6.0 (>5.0)	35.3 (>40)
Sup peron. nerve	2.43	3.4 (>1.0)	45.3 (>40)	2.62	2.4 (>1.0)	47.4 (>40)
Sural nerve	/	/ (>1.0)	/ (>40)	/	/ (>1.0)	/ (>40)
**Others**	F-wave Lat, ms	F-wave Fre, %	H-reflex Lat, ms	F-wave Lat, ms	F-wave Fre, %	H-reflex Lat, ms
	38.4 (<33)	100 (>50)	40.9	37.8 (<33)	100 (>50)	40.8

DML, distal motor latency; CMAP, compound motor action potential; CV, conduction velocity; SNAP, sensory nerve action potential; Lat, latency; Fre, frequency. Reference values at relevant age in our laboratory were shown in brackets.

## Discussion

NIID is a heterogeneous disease entity with variable clinical manifestations involving cognitive, motor, sensory and autonomic systems (1). Similar to other neurodegenerative diseases, most symptoms of NIID progress chronically. Intriguingly, some patients have the paroxysmal phenotype of encephalitic episodes usually presented with disturbance of consciousness, stroke-like episodes, and generalized convulsions accompanied by autonomic dysfunction (5). However, no cases have yet been reported with paroxysmal sensory neuropathy-like onset symptoms to our knowledge. In this study, we reported such a NIID case with recurrent numbness attacks as the main complaint with extensively subclinical-impaired findings of electrophysiology and absent “cortical line sign” on DWI of MRI.

The sensory episodes in the present case were distinct from previously reported paroxysmal phenotype which usually involves diffused cortical lesions with unconsciousness, hemiplegia, aphasia, or epileptic seizures (6–9). Related hyperintensity, edematous areas, and perfusion alterations in the brain imaging have been reported to characterize those typical encephalitic attacks (3). Rather, our patient had very slight paresthesia in the upper extremities with atypical MRI findings of NIID which could not explain the symptom. Also, the distribution of the numbness is reminiscent of that in peripheral neuropathy and led us to decode the diagnosis from the electrophysiological studies.

This case provides a representative example of the mismatching feature between the subjective manifestation and objective examination in NIID, similar to other hereditary or neurodegenerative diseases (10, 11). Patients usually have very mild discomforts, which do not reconcile with the severity of their clinical examinations. Although electrophysiological tests revealed extensive lesions involving the lower extremities and the motor nerves, the patient has never complained of such discomfort throughout the course. Previous studies showed that 44.4–72.2% of NIID patients had clinically overt symptoms of peripheral neuropathy, while the overall reported incidence of NIID-related peripheral neuropathy could be as high as 91.8% (1, 5, 12). This indicated the prevalent involvement of sub-clinical peripheral neuropathy in NIID, which is consistent with our patient. Overall, predominate demyelinating with mild axonal impairments are the primary electrophysiological pattern of NIID. The demyelinating impairments are homogeneous, extensive, and slight which presented with comprehensively decreased nerve conduction velocity and prolonged latency of F-waves and H-reflexes (13). This unique electrophysiological pattern provides subtle clues for differential diagnosis of NIID with other peripheral neuropathies from the functional perspective (14). The homogeneity of lesions with no conduction blocks or dispersions is distinguished from the immune-mediated neuropathy like chronic inflammatory demyelinating polyneuropathy (15). The relatively slight lesions are different from the ganglionopathy with paraneoplastic or other neurodegenerative etiologies (16). Although the extensive involvement of all extremities mimicking metabolic or toxic neuropathy, the minor lesions of axons are unsupported (17). Additionally, the plexus MRI and the sural nerve biopsy of our patient confirmed the representative lesions of NIID from the structural perspective. Further studies are needed to clarify the more susceptible role of the toxicity to Schwann cells than the neurons in the pathological mechanism of peripheral neuropathy in NIID (2).

The mechanisms of paroxysmal symptoms in NIID have not been fully understood yet. According to previous studies, we proposed three possible hypotheses for the recurrent sensory attacks in our patient. First, previous studies have identified the transient vasospasm and hypoperfusion in NIID leading to stroke-like episodes (7, 18). However, this was inconsistent with the negative MRI and MRA results of our patients. Further imaging of arterial spin labeling (ASL) and transcranial doppler (TCD) might help to identify the relevant impairments. Second, hypoxic encephalopathy with large areas of cytotoxic edema could cause epileptic seizures in NIID (19). However, absence of hyperintensity in DWI suggested no cytotoxic edema in our patient, and the clinical pattern of bilateral numbness with awareness remained and no auras was not common in the encephalitic attack. Indeed, electroencephalography (EEG) examinations are required to further confirm whether the patient has epilepsy or not, which we plan to conduct during the follow-up of our patient for more evidence. Third, as paroxysmal symptoms have been reported in patients with multiple sclerosis, the episodes could be mediated by ephaptic impulses and transmissions reflecting the impaired conductions of nerve fibers with partially demyelinated lesions (20–22). Although this seemed to be supported by the demyelination in both MRI and electrophysiological findings of our patient, further studies are needed to elucidate the pathophysiology of different paroxysmal symptoms in NIID.

Our patient showed a unique clinical symptom and uncommon MRI findings that made it difficult to diagnose him with NIID in the early stage. Initially, we focused on the diagnosis of peripheral neuropathy since the electrophysiological results were similar to those of Charcot–Marie–Tooth (CMT) disease (14). Besides, the positive serum immune electrophoresis indicated the diagnosis of neuropathy associated with IgM monoclonal gammopathy of undetermined significance (MGUS) (23). However, the pathological findings of the sural nerve were not supportive and the manifestation of tremors and cognitive impairment could not be explained. Thus, to further elucidate the case from the perspective of monism, we assume a likely diagnosis of NIID which was eventually confirmed by the skin biopsy and genetic analysis.

In summary, we should consider NIID as a differential diagnosis for the undetermined etiology of sensorimotor neuropathy even lacking the characteristic brain MRI findings in the early stage. The present report demonstrates the electrophysiological characteristics of certain paroxysmal sensory deficits in NIID, which addresses the prevalence of sub-clinical peripheral nerve impairments and advances the clinical spectrum of NIID.

## Data availability statement

The datasets presented in this article are not readily available because of ethical and privacy restrictions. Requests to access the datasets should be directed to the corresponding author.

## Ethics statement

The studies involving human participants were reviewed and approved by the Institutional Ethics Committee of Peking University Third Hospital. The patients/participants provided their written informed consent to participate in this study. Written informed consent was obtained from the individual(s) for the publication of any potentially identifiable images or data included in this article.

## Author contributions

JF and CZ: data collection and drafting the manuscript. GH, XL, and MZ: data evaluation and manuscript revision. YSZ and DZ: pathological test. SZ, YXZ, XH, and SY: data collection. DF: funding, data collection and evaluation, supervision, manuscript revision, and final approval. All authors contributed to the article and approved the submitted version.
